# Microvascular decompression for neurovascular compression syndromes secondary to vertebrobasilar dolichoectasia: a single-center retrospective analysis

**DOI:** 10.3389/fsurg.2025.1668352

**Published:** 2025-10-14

**Authors:** Mauro Alberto Segura-Lozano, Mario Alexis Del Real-Gallegos, Pedro Mendoza-Lemus, Bernardo Tenorio-González, Yael Rodrigo Torres-Torres, Alejandro González-Silva, Octavio Carranza-Rentería, Angel Gabriel Parra-Galván, Aarón Giovanni Munguía-Rodríguez

**Affiliations:** 1 Neurología Segura Medical Center, Hospital Angeles Morelia, Morelia, Mexico; 2 Facultad de Ciencias Médicas y Biológicas, Universidad Michoacana de San Nicolás de Hidalgo, Morelia, Mexico; 3 Escuela de Medicina y Ciencias de la Salud, Instituto Tecnológico y de estudios Superiores de Monterrey, Guadalajara, Mexico; 4 Escuela de Estudios Superiores Interdisciplinarios, Universidad Michoacana de San Nicolás de Hidalgo, Morelia, Mexico

**Keywords:** trigeminal neuralgia, hemifacial spasm, glossopharyngeal neuralgia, vertebrobasilar dolichoectasia, microvascular decompression, interposition, transposition

## Abstract

**Background:**

Vertebrobasilar dolichoectasia (VBD) is a rare cause of neurovascular compression syndromes (NVCS), including trigeminal neuralgia (TN), hemifacial spasm (HFS), and glossopharyngeal neuralgia (GPN). Microvascular decompression (MVD) is the primary surgical treatment; however, VBD-related cases are technically challenging and carry a higher risk of complications.

**Objective:**

To analyze the clinical characteristics, surgical findings, outcomes, and complications of patients with NVCS secondary to VBD treated with MVD.

**Methods:**

A retrospective single-center study was conducted on 68 patients who underwent MVD for VBD-associated NVCS between January 2014 and December 2024. Clinical, imaging, intraoperative, and postoperative data were collected and analyzed. Interposition and transposition techniques were employed according to intraoperative findings.

**Results:**

Among the 68 patients, TN was present in 49 cases (72.1%), HFS in 7 (10.3%), GPN in 4 (5.9%), and combined neuropathies in 8 (11.8%). Complete symptom relief was achieved in 92.5% of TN/GPN cases and 55.6% of HFS. During follow-up (mean = 27.3 months), TN recurred in 5.3% and HFS in 11.1%. Transient complications occurred in 67.6%, of patients, while persistent deficits were reported in 36.8%. Comparative analysis showed that patients with VBD were older (*p* < 0.001), predominantly male (*p* < 0.001), and had a higher prevalence of hypertension (*p* < 0.001) or diabetes (*p* = 0.014) compared to those with classical NVCS.

**Conclusions:**

MVD remains a safe and effective treatment for NVCS caused by VBD. However, the technical complexity of these cases demands meticulous surgical planning and long-term follow-up. Both interposition and transposition techniques yielded favorable outcomes.

## 1 Introduction

Neurovascular compression syndromes (NVCS) are predominantly caused by vascular compression of cranial nerves at their root entry zones (REZ). The most commonly affected nerves include the trigeminal nerve (CN V), facial nerve (CN VII), and glossopharyngeal nerve (CN IX), leading to trigeminal neuralgia (TN), hemifacial spasm (HFS), or glossopharyngeal neuralgia (GPN), respectively (1). In most cases, the offending vessels are arteries such as the superior cerebellar artery (SCA), anterior inferior cerebellar artery (AICA), or posterior inferior cerebellar artery (PICA) (1). However, in rare cases, major vessels such as the vertebrobasilar artery (VBA) may contribute directly or indirectly to neurovascular compression, especially when showing ectasia, elongation, or tortuosity, collectively termed vertebrobasilar dolichoectasia (VBD) (2).

Microvascular decompression (MVD) is the most effective treatment for NVCS refractory to medical therapy, offering the advantage of addressing the underlying etiology without causing nerve damage (3). Despite its efficacy, MVD for VBD-associated NVCS is particularly demanding due to the difficulty of mobilizing this large and tortuous vessel (4). Two main surgical modalities of MVD are employed to address an offending VBA: interposition, the standard approach, which involves placing an artificial or autologous implant between the nerve and vessel; and transposition or retraction, in which the VBA is repositioned away from the nerve. This can be achieved by gentle mobilization or by anchoring the artery to the petrous dura or tentorium. Various materials have been used for this purpose, including Teflon (polytetrafluoroethylene) slings, rolls, pads, or felts; autologous muscle; aneurysm clips; vascular tapes; silicone sheets; Ivalon sponges; dry Gelfoam; polypropylene sutures; titanium plates; and biomedical adhesives (5–7).

The tortuosity and rigidity of the VBA may increase the risk of complications, including injury to cranial nerves or perforating arteries of the brainstem (8). Furthermore, recurrence rates and long-term outcomes remain variable, underscoring the need for further investigation. This study presents a retrospective case series of patients undergoing MVD for TN, HFS, or GPN secondary to VBD. By analyzing clinical characteristics, perioperative management, and outcomes, we aim to enhance the understanding and optimization of treatment strategies for this challenging patient subgroup.

## 2 Materials and methods

### 2.1 Patient population and selection criteria

This retrospective single-center study included patients who underwent MVD for NVCS secondary to VBD at our institution between January 2014 and December 2024. Eligible patients presented with TN, HFS, or GPN, confirmed through clinical evaluation and supported by imaging studies. All patients were considered refractory to medical therapy, defined as inadequate pain control, intolerable adverse effects, or severe drug-related complications associated with first-line medications (typically carbamazepine or oxcarbazepine) administered at therapeutic doses. Patients who initially responded to pharmacological treatment but subsequently experienced disabling relapse were also included. In HFS, refractoriness was defined as insufficient benefit from oral medications, intolerance to therapy, or diminished efficacy after repeated botulinum toxin injections. Patients with idiopathic neuropathies or secondary non-vascular etiologies, such as intracranial tumors or demyelinating diseases, were excluded.

Detailed data were collected for each patient, including demographics (age, sex); clinical features (symptom duration, affected side, involved trigeminal branches, pain characteristics); comorbidities (hypertension, diabetes mellitus, dyslipidemia, obesity); previous treatments (MVD, radiofrequency thermocoagulation, glycerol rhizotomy, stereotactic radiosurgery, botulinum toxin); imaging findings; intraoperative observations (offending vessels, compression severity, arachnoid adhesions, presence of atherosclerosis, suprameatal tubercle size); surgical technique (interposition or transposition); postoperative outcomes; complications; and follow-up duration. Written informed consent was obtained from all patients.

### 2.2 Imaging studies

All patients underwent preoperative MRI, including high-resolution 3D sequences (3D-FIESTA/CISS). When available, magnetic resonance angiography (MRA) or digital subtraction angiography (DSA) were performed to assess vascular tortuosity and anatomical course. Imaging studies were focused on the pontine region and the trajectory of the cranial nerves. Initial reports from external centers were retrieved from medical records, and, prior to surgery, all studies were independently re-evaluated by our institutional neuroradiologists blinded to the clinical data.

### 2.3 Surgical procedures

All surgeries were performed by the same senior neurosurgeon via a retrosigmoid craniotomy. Patients were placed in the lateral decubitus position with approximately 45° of head rotation to allow optimal exposure of the cerebellopontine angle. Key surgical steps included dural opening with gradual cerebrospinal fluid drainage to promote passive cerebellar relaxation, gentle retraction of the cerebellum along the tentorial and petrosal surfaces to expose the cisternal anatomy and cranial nerves, and careful dissection of arachnoid adhesions when present.

**Figure 1:**
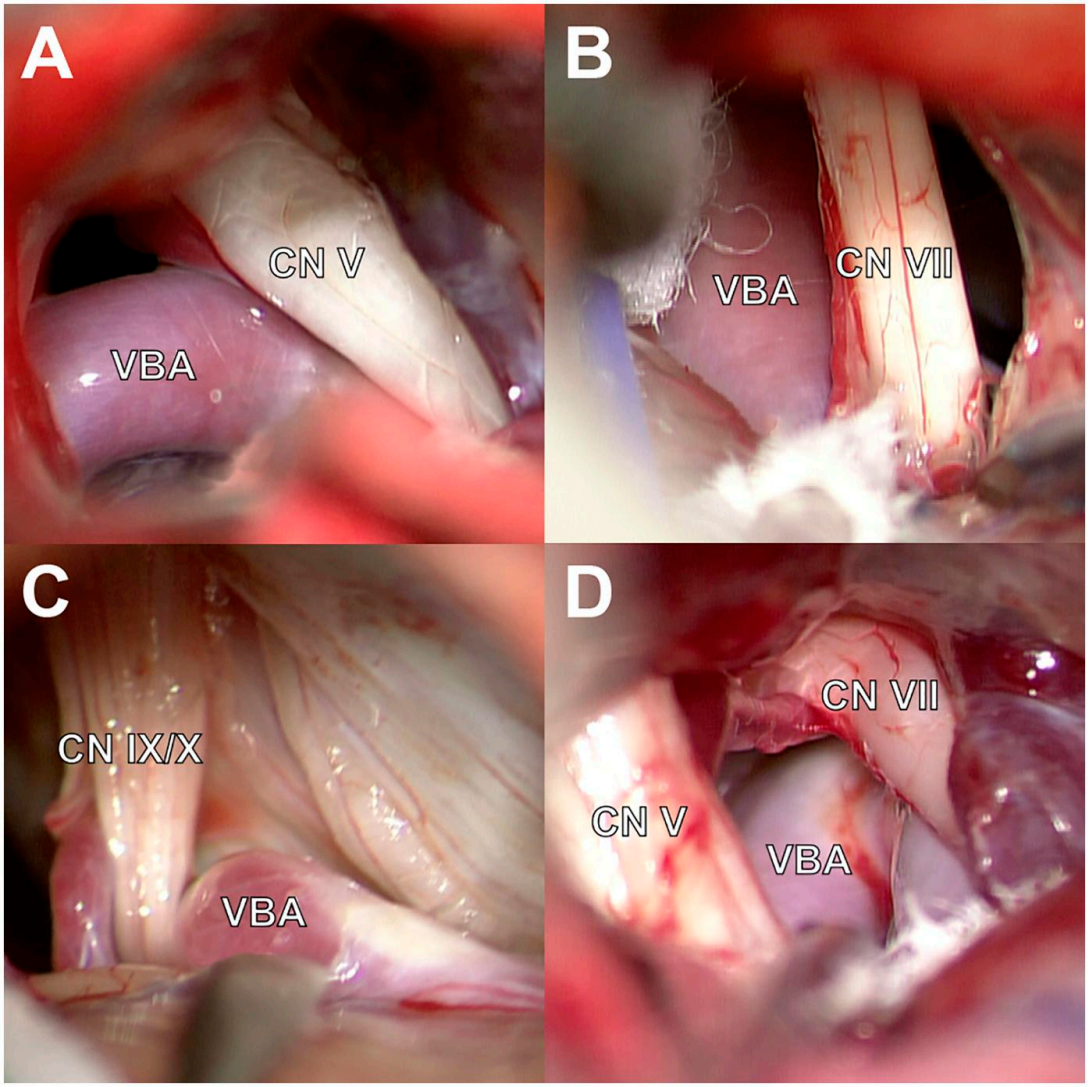
Intraoperative findings during MVD for TN, HFS, and GPN caused by VBD. **(A)** VBD compressing the CN V. **(B)** VBA in contact with the CN VII. **(C)** Contact between VBA and the CN IX. **(D)** Multi-cranial nerve compression by the VBA and surrounding vessels with CN V and CN VII.

**Figure 2:**
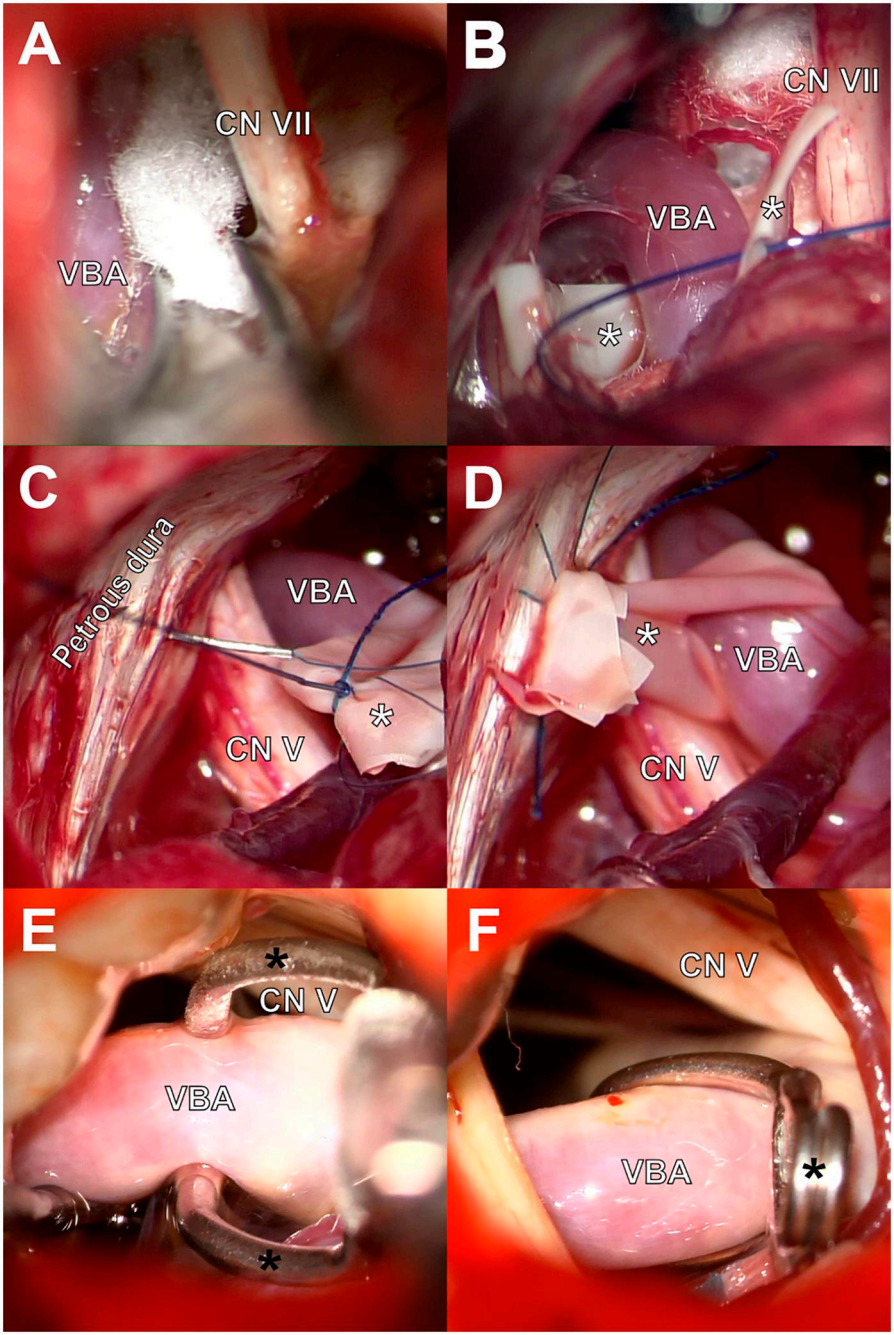
Surgical techniques for decompression of the VBD-related cranial nerve compressions. **(A)** Interposition of shredded Teflon felt between VBA and CN VII. **(B)** Preparation of transposition using a latex sling encircling the VBA. **(C,D)** Fixation of both ends of the sling to the petrous dura using polypropylene sutures. **(E,F)** Transposition of the VBA using a fenestrated aneurysm clip sutured to the tentorium.

The offending vessels, including the VBA, were managed using either interposition or transposition techniques, with the choice of strategy determined intraoperatively according to the findings and complexity of the neurovascular conflict (Figure 1). In relatively straightforward cases, interposition was performed by placing an adequate amount of shredded Teflon felt between the VBA and the affected nerve, serving as a mechanical barrier to relieve compression (Figure 2A). Transposition/retraction was required under the following circumstances: (a) if significant pulsatile transmission persisted after adequate padding; (b) when Teflon padding alone was unstable or insufficient to maintain the VBA away from the nerve; (c) Szapiro's grade III compression; (d) when the conflict involved not only the VBA but also additional vessels requiring multiple Teflon implants; (e) when arterial stiffness or atherosclerosis of the VBA allowed its safe mobilization.

**Figure 3:**
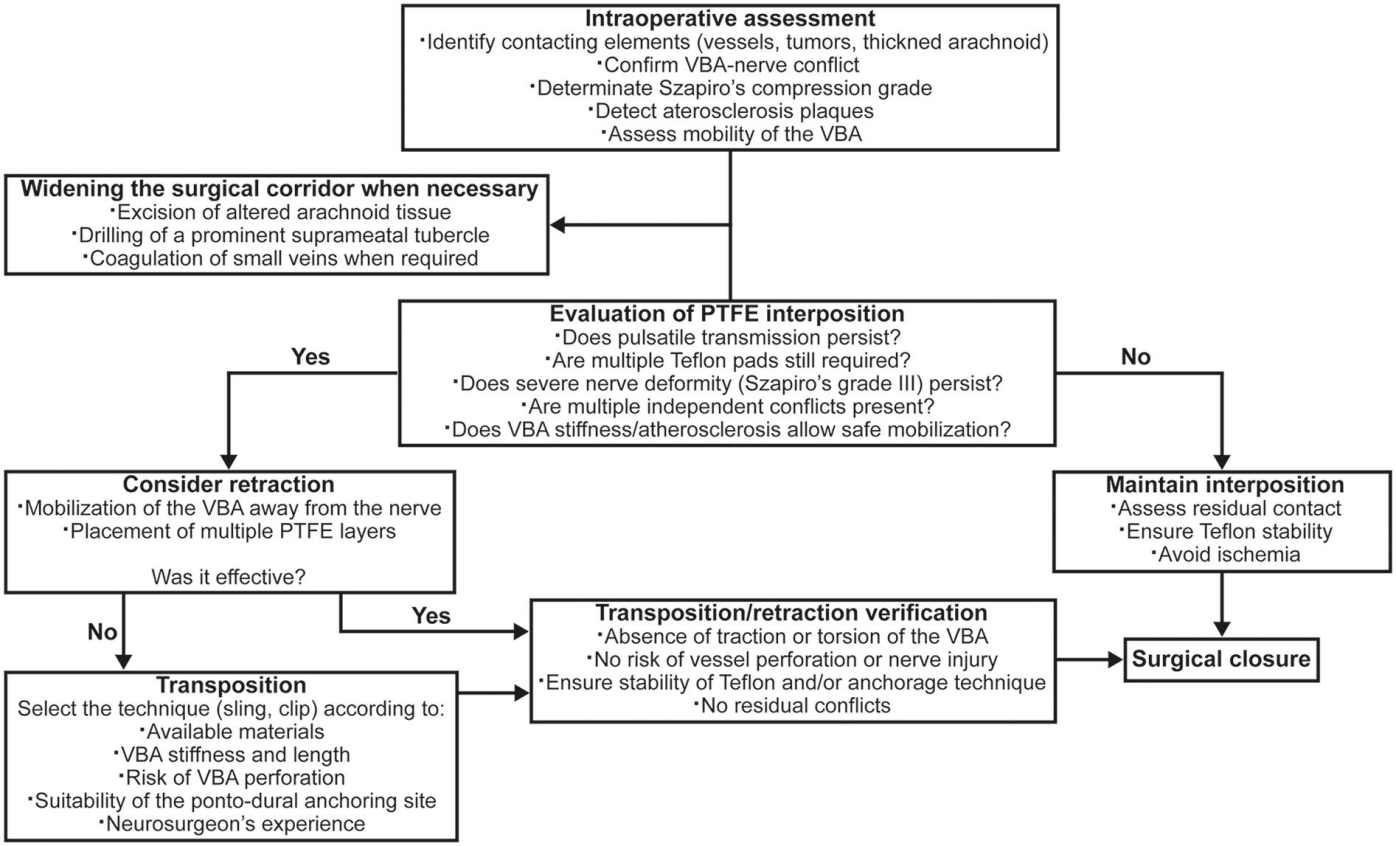
Flowchart of the intraoperative decision-making algorithm for interposition versus transposition during MVD in VBD-related NVCS. VBA, vertebrobasilar artery. Szapiro's grade III: the offending vessel causes nerve deformation, impression, or indentation.

The transposition/retraction procedure involved the gentle displacing of the VBA to a location sufficiently distant from the nerve. In some cases, retraction was achieved by placing additional Teflon pads; while in others, the artery was repositioned using a stitched sling technique. For this approach, a sterile latex band of appropriate length was looped around the VBA, leaving a tab to anchoring (Figure 2B). Both ends of the sling were secured to the petrous dura or the tentorium using polypropylene sutures (Figures 2C,D). When necessary, biomedical fibrin glue was applied for additional fixation. In one case, transposition was accomplished by passing the VBA through the fenestration of a Sugita titanium aneurysm clip, which was subsequently sutured to the tentorium at a lateral position sufficient to maintain the vessel away from the affected nerve (Figures 2E,F). After successful isolation of the offending VBA, the surgical field was reevaluated to identify other neurovascular conflicts. Additional arterial loops and high-flow veins were managed by interposition, whereas small veins were coagulated and sectioned when they were not critical tributaries. A flowchart illustrating the intraoperative decision-making algorithm for selecting interposition or transposition during MVD in VBD-related NVCS is shown in Figure 3.

### 2.4 Intraoperative observations

**Figure 4:**
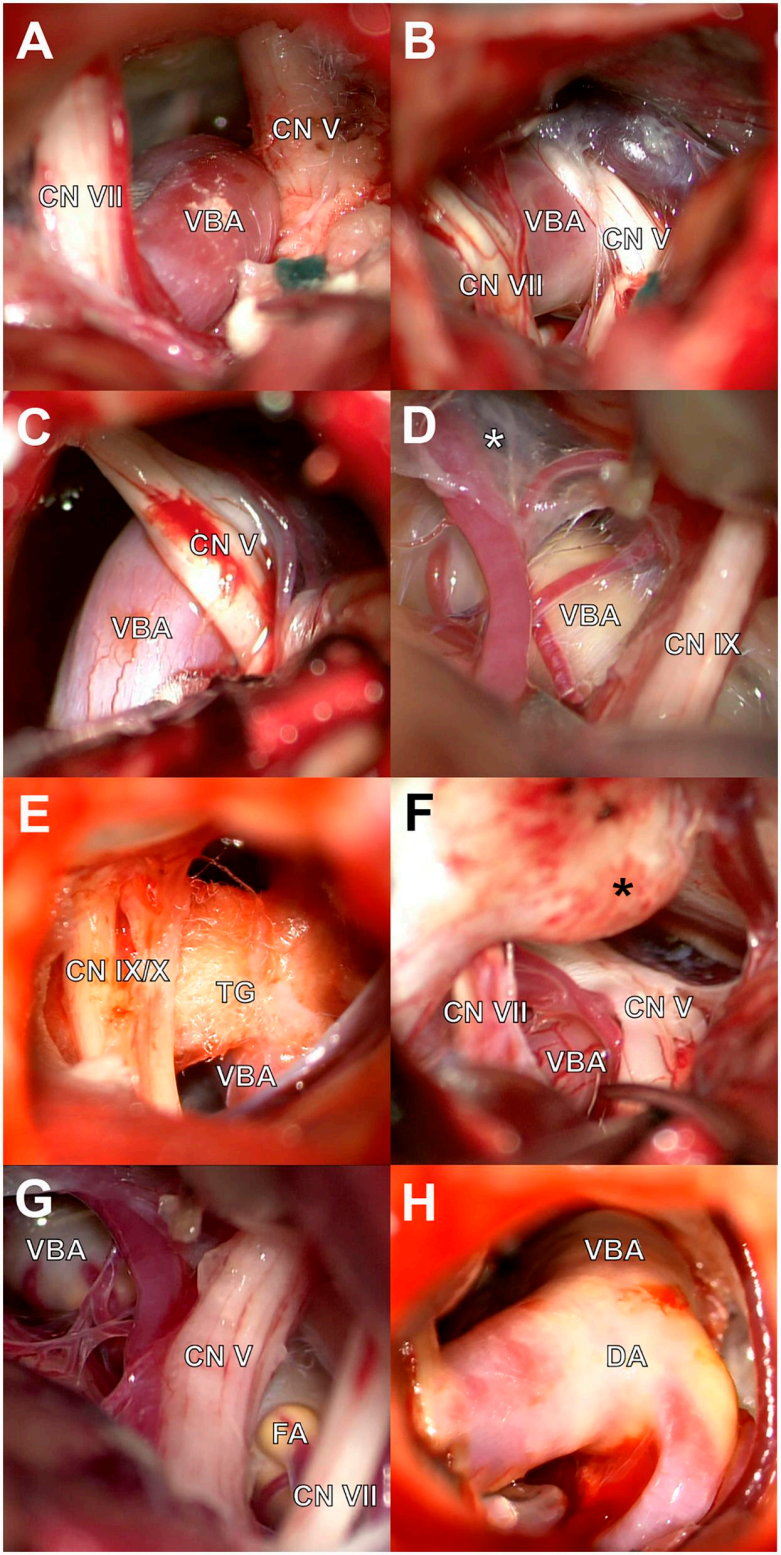
Intraoperative anatomical findings in VBD-associated neurovascular conflicts. **(A)** Grade I compression: VBA in contact with the CN VII without distortion. **(B)** Grade II compression: evident displacement of the CN V and VII by the VBA. **(C)** Grade III compression: CN V deformation due to VBA compression. **(D)** Altered arachnoid tissue (white asterisk) with fibrous consistency. **(E)** Teflon granuloma (TG) causing recurrent compression. **(F)** Prominent suprameatal tubercle (black asterisk) requiring drilling. **(G,H)** Focal (FA) and diffuse (DA) atherosclerotic plaques identified on the VBA.

The severity of vascular compression was assessed using Szapiro's classification (9), which defines three grades: grade I, the vessel is in contact with the nerve without obvious signs of compression (Figure 4A); grade II, clear compression with or without displacement of the nerve (Figure 4B); and grade III, the offending vessel causes nerve deformation, impression, or indentation (Figure 4C).

When arachnoid alterations were encountered, the tissue was dissected and classified based on consistency as either viscous or fibrous (Figure 4D). In reoperated patients, Teflon granulomas were occasionally found as the source of recurrent neurovascular compression (Figure 4E). The suprameatal tubercle classification was according its size: type I (0–1 mm); type II (2–3 mm); and type III (>3 mm) (10) (Figure 4F). Prominent suprameatal tubercles were drilled when necessary. Findings of atherosclerotic plaques were classified as focal (Figure 4G) or diffuse (Figure 4H).

### 2.5 Postoperative evaluation

**Figure 5:**
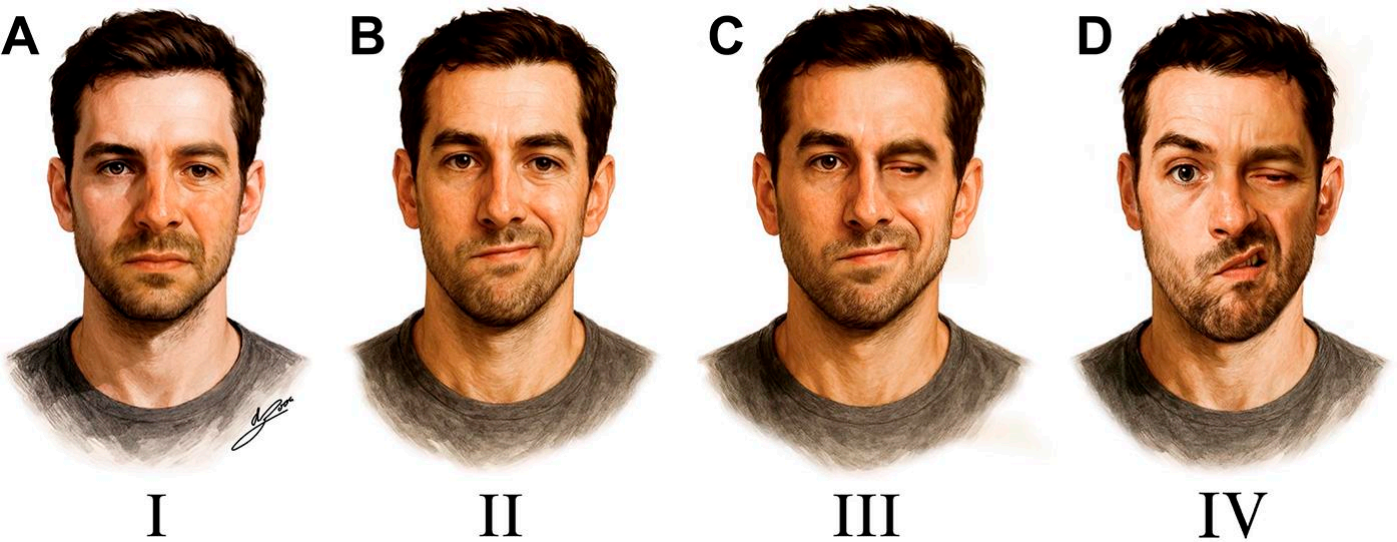
Facial spasm severity grading based on the Samsung medical center (SMC) classification. **(A)** Grade I: spasm confined to the periocular area. **(B)** Grade II: spasm spreads to other ipsilateral facial muscles. **(C)** Grade III: vision interference due to frequent tonic spasms. **(D)** Grade IV: severe facial distortion with tonic contractions impairing eye opening.

Pre- and postoperative pain relief for TN and GPN was assessed using the Barrow Neurological Institute (BNI) Pain Intensity Scale: Class I, no pain and no medication required; Class II, occasional pain without need for medication; Class III, some pain adequately controlled with medication; Class IV, pain not adequately controlled with medication; and Class V, severe pain not relieved by medication (11). Pain recurrence was defined as a return to preoperative pain scores (BNI IV or V) after achieving initial relief. In cases of HFS, the severity of involuntary movements was assessed using the Samsung Medical Center (SMC) grading system (12): Grade I, localized spasm limited to the periocular area; Grade II, involuntary movements spreading to other ipsilateral facial muscles, including the orbicularis oris, zygomaticus, frontalis, and platysma; Grade III, spasms occurring so frequently or with such tonic intensity that they interfere with vision, especially eyelid opening; and Grade IV, disfiguring facial asymmetry with continuous orbicularis oculi contractions, severely impairing eye function (Figure 5). Postoperative outcomes for HFS were categorized as complete relief (CR), defined as the absence of HFS; partial relief (PR), indicating a reduction of more than 50% in symptoms; and no relief (NR), referring to less than 50% improvement or unchanged symptoms. Long-term follow-up was conducted via telephone or in-person interviews, and in cases where direct contact was not possible, data from the most recent clinical evaluation were used.

### 2.6 Statistical analysis

Data analysis was performed using SPSS version 23 (IBM Corp., Armonk, NY, USA). Continuous variables were expressed as mean ± standard deviation, and categorical variables as frequency and percentage. Comparisons between VBD-associated and classical NVCS cases were performed using the chi-square or Fisher's exact test for categorical data, and the Mann–Whitney *U* test for non-parametric continuous variables. The postoperative maintenance of symptom-free status was evaluated in the TN, HFS, and GPN groups using the Kaplan–Meier method and compared with the log-rank test. A *p*-value < 0.05 was considered statistically significant.

## 3 Results

### 3.1 Demographics and clinical characteristics

A total of 68 patients with NVCS secondary to VBD were included (Table 1). VBD-related neuropathies included TN alone in 49 cases (72.1%), HFS in 7 (10.3%), GPN in 4 (5.9%), TN combined with GPN in 6 (8.8%), and TN combined with HFS in 2 (2.9%). The incidence of neurovascular conflict involving VBD was 6% (*n* = 68) among all our patients who underwent surgery for NVCS caused by vascular compression during the 11-year study period (*n* = 1,134). Among all our patients who presented with TN symptoms (*n* = 1,041), VBA was compressing the CN V in 51 cases (4.9%). In HFS cases (*n* = 106), this artery was in contact with the CN VII in 9 (8.5%), while for GPN (*n* = 67), VBA was identified in 11 patients (16.4%). Additionally, among all those cases, combined compressive neuropathies were identified in 80 patients, with VBA involvement observed in 8 of them (10%).

**Table 1:** Baseline clinical characteristics of the cohort.

Case	Diagnosis	Age (ys)	Sex	History of symptoms (ys)			Affected side			Medical history	BMI Category	CN V Affected branches	Type of pain	Previous Treatment
				TN	HFS	GPN	TN	HFS	GPN					
1	TN	55	F	3			L			DLP	OW	V1 + V2	E, B	
2	TN	74	F	3			R			DLP, HT, Cardiomegaly	OW	V2	E	PGR x3
3	TN	37	M	9			R			T2DM	OW	V1 + V2 + V3	E	MVD
4	GPN	65	F			20			L	HTN, T2DM, HT	OW	–	E, B	
5	TN	53	F	3			L			HTN, T2DM, DLP	OW	V1 + V2 + V3	E	
6	TN	53	M	2.5			L			HTN, T2DM	OB II	V2 + V3	E	
7	TN	58	M	2.3			R			–	NW	V1 + V2 + V3	E	
8	TN	61	M	2			R			DLP	OB I	V3	E	
9	GPN	36	F			2			R	–	OW	–	E	
10	TN	72	M	9			L			HTN, T2DM	OB I	V2 + V3	E	RT x2
11	TN	55	M	21			L			HTN, T2DM	OB II	V2 + V3	E	
12	TN	68	F	7			R			HTN	OB I	V1 + V2 + V3	E, B, S	MVD TN
13	TN	28	M	13			L			HTN	OB II	V2	E, B, S	RT x3
14	GPN	54	M			10			L	–	OW	–	E	
15	TN	59	M	24			R			–	OW	V1 + V2	E	
16	TN	52	F	4.5			R			HTN, DLP	OB I	V1 + V2 + V3	E, B	
17	TN	59	F	15			R			HTN, T2DM	OB I	V1 + V2 + V3	E	PGR, RT
18	TN	48	M	18			R			–	OW	V1 + V2 + V3	E	
19	TN	77	F	13			R			Epilepsy	OW	V1 + V2 + V3	E	
20	TN	74	M	7			L			–	NW	V2 + V3	E	
21	TN	71	F	12			R			HTN, T2DM	OW	V2	E, B	
22	TN + GPN	37	M	4		3	L		L	HTN	OB I	V3	E	
23	TN	65	F	2			R			HTN	OW	V2	E	
24	TN + GPN	51	M	16		10	R		R	–	OW	V3	E, B, S	PGR x4
25	TN	77	F	4			R			HTN	OW	V2 + V3	E	
26	TN + HFS	56	M	20	5		R	R		HTN	OW	V2	E	BT
27	TN	68	F	10			R			HTN	OW	V1 + V2 + V3	E	
28	TN	47	M	0.42			R			HTN	OW	V2	E	
29	TN	67	M	4			L			–	OW	V1 + V2	E, B	
30	HFS	55	F		12			L		HTN, Epilepsy	OB II	–	–	BT
31	TN + HFS	53	F	10	9		L	L		HTN	OB III	V2 + V3	E, B	
32	TN + GPN	65	M	10		20	R		R	HTN, T2DM	OB I	V3	E, B	
33	HFS	48	M		7			L		HTN	OW	–	–	
34	TN	71	F	5			L			T2DM, DLP	OW	V1 + V2 + V3	E, B, S	
35	TN	62	M	5			R			HTN, T2DM, DLP	OB I	V3	E	
36	HFS	53	M		5			L		HTN, T2DM	OW	–	–	BT
37	TN	60	M	2.5			R			HTN, HT	OW	V1 + V2 + V3	E, B	
38	GPN	60	M			9			L	HTN	OW	–	E, B	
39	TN	57	M	5			L			HTN	OB I	V2 + V3	E	
40	TN	64	M	7			L			–	OB III	V3	E, B	RT
41	TN + GPN	71	F	12		0.5	R		R	HTN, DLP	OW	V1	E, B	
42	HFS	63	M		7			L		HTN, T2DM	OB I	–	–	BT
43	TN	63	M	7			L			–	OB I	V3	E	RT x2
44	TN	79	M	17			L			T2DM	OW	V1 + V2 + V3	E, B, S	PGR, RT
45	TN	68	M	10			L			HTN, T2DM, DLP	NW	V2	E, S	
46	TN	69	M	9			L			HTN	OW	V1 + V2 + V3	E, B	
47	TN	79	F	2			L			HT	OB I	V2	E	
48	HFS	30	M		6			L		–	OW	–	–	BT
49	TN	55	M	10			L			–	OW	V2 + V3	E, B	
50	TN	69	M	0.67			R			DLP	OW	V3	E, S	
51	TN	67	F	18			R			HTN, DLP	OB I	V1 + V2 + V3	E	
52	HFS	62	F		0.48			L		HTN, T2DM, DLP	OW	–	–	BT, MVD TN
53	TN	68	M	4.5			L			HTN, DLP	OW	V2 + V3	E	
54	TN	42	M	2			R			–	OW	V2 + V3	E	PGR
55	HFS	43	M		4			L		–	OW	–	–	
56	TN	79	F	2.5			R			HTN	NW	V1 + V2	E	
57	TN	58	M	6			R			HTN	OB I	V1 + V2	E	
58	TN + GPN	63	M	8		2	L		L	T2DM, Renal insufficiency	OW	V2 + V3	E, B	SRS
59	TN	72	M	2			L			–	OB I	V2 + V3	E	RT
60	TN + GPN	45	M	21		10	R		R	–	OB II	V2	E	RT
61	TN	73	F	4			L			HTN, T2DM, HT	OW	V3	E	RT
62	TN	64	M	11			L			HTN	OB I	V2 + V3	E	MVD
63	TN	73	F	12			L			HTN	OW	V2 + V3	E	
64	TN	52	M	0.5			L			–	OW	V2 + V3	E	
65	TN	83	F	1.9			L			DLP	OW	V2	E	MVD
66	TN	63	M	5			L			T2DM, Liver failure	OB I	V2 + V3	E, B	
67	TN	66	M	2			L			HTN, T2DM	OB II	V2 + V3	E	
68	TN	72	F	13			R			T2DM	OB I	V1 + V2 + V3	E, B	RT x6

TN, trigeminal neuralgia; HFS, hemifacial spasm; GPN, glossopharyngeal neuralgia; F, female; M, male; HTN, hypertension; T2DM, type 2 diabetes mellitus; DLP, dyslipidemia; HT, hypothyroidism; NW, normal weight; OW, overweight; OB, obesity; V1, ophthalmic branch of the CN V; V2, maxillary branch of the CN V; V3, mandibular branch of the CN V; E, electric; B, burning; S, stabbing; PGR, percutaneous glycerol rhizotomy; MVD, microvascular decompression; RT, radiofrequency thermocoagulation; BT, botulinum toxin; SRS, stereotactic radiosurgery.

The mean age of patients with VBD-related NVCS was 60.5 ± 12.1 years (range: 28–83), with 43 males (63.2%) and 25 females (36.8%). The median symptom duration was 8 ± 6.1 years (range: 5 months to 24 years), considering the nerve with the longest clinical history in cases with multiple nerve involvement. Left-sided symptoms were more common (39 patients, 57.4%). The most frequent comorbidity was hypertension (38 cases, 55.9%), followed by type 2 diabetes mellitus (21, 30.9%), dyslipidemia (14, 20.6%), hyperthyroidism (5, 7.4%), and other conditions (5, 7.4%). Only 4 patients (5.9%) had normal weight, while 38 (55.9%) were overweight; grade I obesity was observed in 18 patients (26.5%), grade II in 6 (8.8%), and grade III in 2 (2.9%) (Table 1).

Among TN patients, the most commonly affected trigeminal branches were: V2 + V3 (17 cases, 29.8%), V1 + V2 + V3 (15, 26.3%), V2 alone (10, 17.2%), V3 alone (9, 15.8%), V1 + V2 (5, 8.8%), and V1 alone (1, 1.8%). All 61 patients with TN or GPN reported electric shock-like pain. In 23 patients (37.7%), two or more types of neuropathic pain coexisted; burning pain was described in 21 cases (34.4%) and stabbing pain in 7 (11.5%) (Table 1).

Previous treatments included MVD (5 patients, 7.4%), radiofrequency thermocoagulation (10, 14.7%), percutaneous glycerol rhizotomy (5, 7.4%), and stereotactic radiosurgery (1, 1.5%). Among HFS cases, botulinum toxin was repeatedly administered in 6 of 9 patients (66.6%) (Table 1).

### 3.2 Image findings

**Figure 6:**
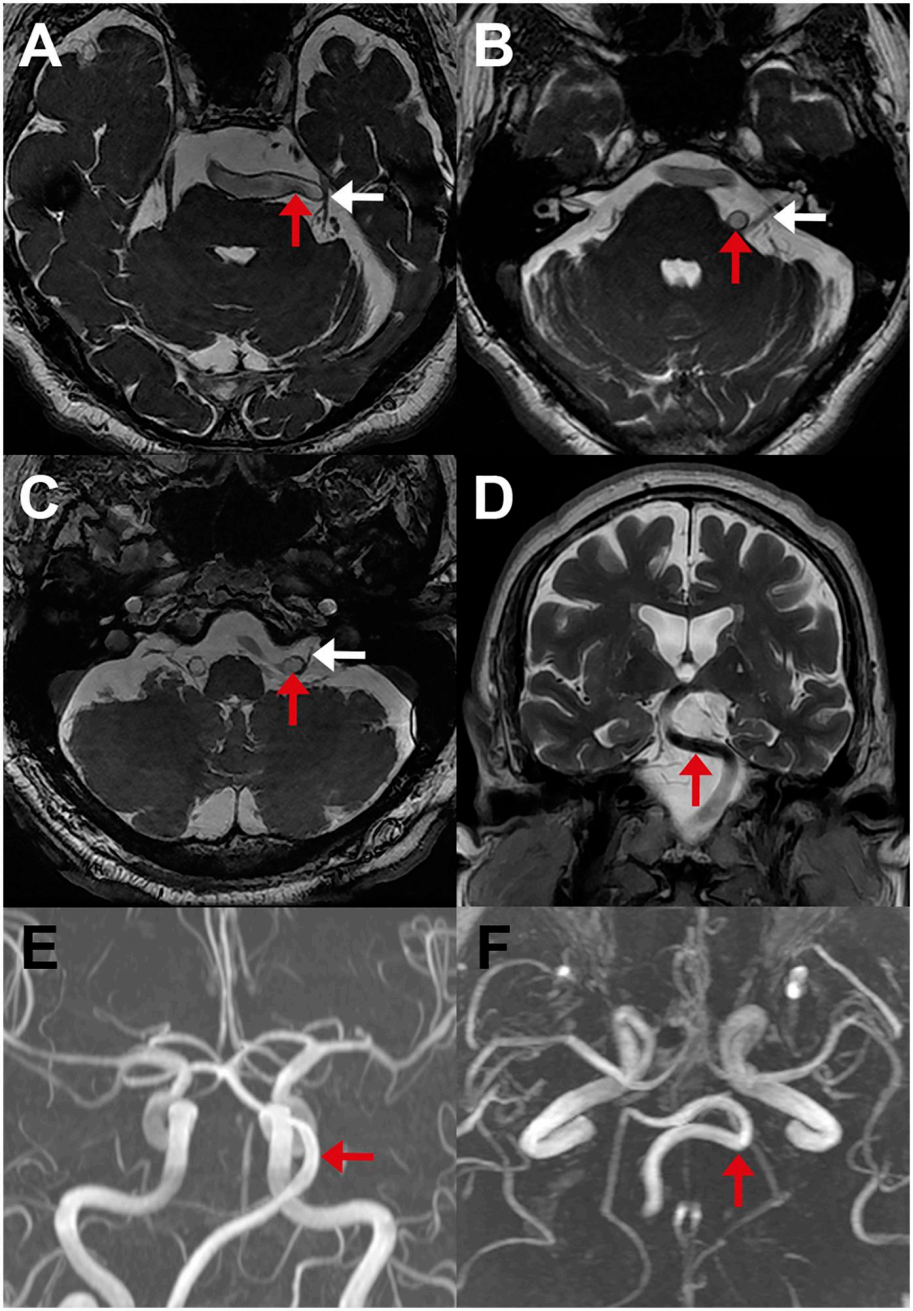
Preoperative neuroimaging findings in patients with cranial nerve compression due to VBD. **(A–C)** MRI 3D-FIESTA sequences showing compression of the CN V **(A)**, CN VII **(B)**, and CN IX **(C)** by the VBA (red arrows); corresponding nerves are indicated with white arrows. **(D)** Coronal MRI suggesting displacement of the brainstem by the VBA (red arrow). **(E,F)** Axial and coronal MR angiography demonstrating the tortuous course of the VBA (red arrows).

Reports from external neuroradiologists using high-resolution MRI sequences (3D-FIESTA or 3D-CCIS) showed concordance with intraoperative identification of VBD in 43 patients (63.2%) (Table 2). However, when imaging was reviewed by our neuroradiologists, VBD was identified in all cases. Representative MRIs are shown in Figure 6 for TN (Figure 6A), HFS (Figure 6B), and GPN (Figure 6C). In a subset of patients, DSA or MRA was also performed (Figures 6E,F), providing valuable information on VBA tortuosity and course, which aided in preoperative planning and selection of the appropriate decompression strategy.

**Table 2:** Neuroimaging and intraoperative findings.

Case	VBA-CN Contact in MRI	Conflicting arteries			Conflicting veins			VBA—Szapiro's classification.			Altered Arachnoid consistency	Atherosclerosis in VBA	Suprameatal tubercle	Surgical technique
		CN V	CN VII	CN IX	CN V	CN VII	CN IX	CN V	CN VII	CN IX				
1	No	VBA						II			Fibrous			Interposition
2	No	VBA						III			Fibrous			Interposition
3	No	VBA			Petrous			III						Interposition
4	No			VBA						II	Fibrous			Interposition
5	Yes	VBA						III			Fibrous			Interposition
6	Yes	VBA, SCA			Afferent to SPVC			III			Viscose			Interposition
7	No	VBA						III				Diffuse		Interposition
8	Yes	VBA, SCA			Pontine			III			Fibrous			Interposition
9	No			VBA						II	Viscose			Interposition
10	Yes	VBA						III			Fibrous	Focal		Interposition
11	Yes	VBA, SCA, AICA			Unnamed			II			Viscose			Interposition
12	No	VBA						II			Fibrous			Interposition
13	No	VBA, SCA						II			Fibrous			Interposition
14	No			VBA						I	Fibrous			Interposition
15	Yes	VBA, SCA						I			Viscose			Interposition
16	No	VBA			Afferent to SPVC			II			Viscose			Interposition
17	Yes	VBA, AICA						I			Fibrous*			Interposition
18	Yes	VBA			Pontine			I			Viscose			Interposition
19	No	VBA, SCA						II			Viscose			Interposition
20	Yes	VBA			Petrous			I			Viscose			Interposition
21	No	VBA, SCA, AICA						II			—			Interposition
22	Yes	SCA		VBA						II	Viscose			Interposition
23	Yes	VBA						III			Viscose			Interposition
24	No			VBA	Unnamed					II	Viscose			Interposition
25	No	VBA			Pontine			II			Viscose			Interposition
26	Yes	VBA	VBA					III	II		Viscose			Interposition
27	Yes	VBA, AICA						I			Viscose			Interposition
28	Yes	VBA			Afferent to SPVC			III			Viscose			Interposition
29	Yes	VBA, SCA						II			Viscose			Interposition
30	Yes		VBA						II		Viscose			Interposition
31	Yes	SCA	VBA		Unnamed				II		Viscose	Diffuse		Interposition
32	Yes	SCA		VBA	Pontine					III	Viscose	Diffuse		Interposition
33	Yes		VBA, AICA			Pontine			II		Viscose	Diffuse		Interposition
34	No	VBA, SCA, AICA			Pontine, Afferent to SPVC			II			Viscose			Interposition
35	No	VBA, SCA			Pontine			II			Viscose	Focal		Interposition
36	Yes		VBA						III		Viscose			Interposition
37	No	VBA, AICA			Pontine			I			Viscose	Focal		Interposition
38	No			VBA, PICA						I	Viscose			Interposition
39	Yes	VBA, SCA			Afferent to SPVC			I			Fibrous			Interposition
40	Yes	VBA, SCA, AICA			Pontine			I			Viscose			Interposition
41	No	SCA		VBA, PICA						II	Viscose			Interposition
42	Yes		VBA, AICA						II		Viscose			Interposition
43	Yes	VBA						II			Fibrous			Interposition
44	Yes	VBA, SCA			Afferent to SPVC			III			Viscose			Interposition
45	Yes	VBA, SCA				Pontine		II			Viscose			Interposition
46	Yes	VBA						II			Viscose			Interposition
47	Yes	VBA, SCA, AICA						II				Diffuse		Interposition
48	No		VBA, AICA						II		Viscose		Grade III	Interposition
49	Yes	VBA, SCA						III			Fibrous			Interposition
50	No	VBA						II			Fibrous			Interposition
51	No	VBA, SCA						I			Fibrous		Grade III	Interposition
52	Yes		VBA						II		Fibrous*			Interposition
53	Yes	VBA, SCA			Afferent to SPVC			III			Viscose			Transposition
54	Yes	VBA			Afferent to SPVC			III			Viscose			Interposition
55	No		VBA		Pontine				II		Fibrous			Interposition
56	Yes	VBA						III			Viscose		Grade III	Interposition
57	No	VBA, AICA			Unnamed			III			Fibrous			Interposition
58	Yes	VBA, SCA		VBA				III		II	Viscose			Interposition
59	Yes	VBA						II			Viscose			Interposition
60	Yes	AICA		VBA	Pontine					II	Fibrous			Interposition
61	Yes	VBA, SCA						III			Fibrous		Grade III	Interposition
62	Yes	VBA						II				Diffuse		Interposition
63	Yes	VBA			Pontine			III			Viscose			Transposition
64	No	VBA						III			Viscose			Transposition
65	Yes	VBA						I			Fibrous*	Diffuse		Interposition
66	Yes	VBA			Pontine			III				Diffuse		Transposition
67	Yes	VBA, SCA						III						Interposition
68	Yes	VBA			Afferent to SPVC			III			Fibrous			Interposition

VBA, vertebrobasilar artery; SCA, superior cerebellar artery; AICA, anterior inferior cerebellar artery; PICA, posterior inferior cerebellar artery; SPVC, superior petrosal venous complex; Pro, proximal portion of the nerve; Mid, middle portion; Dis, distal portion; T, transposition; I, interposition. Asterisk (*) indicates postoperative fibrous arachnoid.

### 3.3 Surgical findings

Intraoperatively, the VBA was involved in all cases. It was identified as the sole offending vessel in 34 patients (50%), while multi-vessel compression was observed in the remaining cases. Arterial-only compression occurred in 40 patients (58.8%), while mixed (arterial and venous) compression was identified in the remaining 28 patients (41.2%). Most common associated offending arteries included the SCA in 25 patients (36.8%) and AICA in 12 (17.6%); the PICA was observed only in two patients with GPN (2.9%). Among venous offenders, the most frequently involved vessels were: pontine vein (14, 20.6%), afferent veins to the superior petrosal venous complex (9, 13.2%), unnamed veins (5, 5.9%), and petrous veins (2, 2.9%) (Table 2). Notably, in two cases, the VBA compressed two cranial nerves simultaneously.

According to Szapiro's classification, the severity of compression by VBA was grade I in 12 patients (17.6%), grade II in 33 (48.5%), and grade III in 25 (36.8%). Altered arachnoid tissue was found in 61 patients (89.7%), with viscous consistency in 39 (57.4%) and fibrous in 22 (32.4%); three cases (4.4%) were probably related to postoperative arachnoid changes. In parallel, focal or diffuse atherosclerotic plaques were found in 11 cases (16.1%). Additionally, prominent suprameatal tubercles (type III) were observed in 4 patients (5.9%), and drilling was required in two cases to expand the surgical corridor and facilitate MVD. The surgical strategies employed consisted of Teflon interposition in 64 cases (94.1%) and VBA transposition in four cases (5.9%) using the sling technique or a fenestrated aneurysm clip (Table 2).

### 3.4 Treatment results

Immediate postoperative outcomes among patients with TN and GPN included complete pain relief (BNI I) in 92.5%, partial pain relief (BNI II–III) in 7.5% and no patients experienced persistent pain (BNI IV–V). At last follow-up, recurrence of TN was observed in 3 patients (5.3%) at an average of 17.6 ± 14.7 months postoperatively, all of whom responded to medical management. Notably, one patient who initially underwent MVD for TN developed ipsilateral HFS 5.8 years later.

For HFS, five patients (55.6%) experienced complete resolution, and four (44.4%) had partial relief immediately after surgery. Among patients who exhibited partial relief, one patient had complete recurrence two months postoperatively, while another reported delayed complete resolution six months after surgery. The remaining patients were reported to be spasm-free at last follow-up (Table 3).

**Table 3:** Surgical outcomes and postoperative follow-up.

Case	Pre-surgical BNI/SMC			Immediate postoperative outcome			Last follow-up outcome			Immediate complications	Long-term complications	Follow-up (months)
	TN	HFS	GPN	TN	HFS	GPN	TN	HFS	GPN			
1	IV			I			II			Conjunctival injection, Epiphora	None	60
2	V			I			I			None	None	24
3	V			I			I			None	None	82
4			IV			I			II	None	Dysphonia	39
5	V			I			I			Hearing impairment, Grade II FP	Hearing impairment	36
6	V			I			I			None	None	48
7	V			II			IV			None	Recurrence (1 month)	6
8	V			I			I			Vertigo, Hearing impairment	Hearing impairment	36
9			V			I			I	None	None	48
10	V			I			III			Nasal fistula	Recurrence (29 months)	66
11	V			I			I			Grade II FP	None	72
12	IV			I			I			None	Xerophthalmia	6
13	V			I			I			None	None	32
14			V			I			I	None	None	45
15	V			I			I			Grade I FP	Grade I FP	24
16	V			II			II			Anisocoria, Eyelid ptosis	None	52
17	V			I			I			None	None	64
18	V			I			I			None	Hearing impairment	12
19	V			I			I			None	None	18
20	V			I			I			Paresthesia	None	6
21	V			I			I			None	None	36
22	V		V	I		I	II		I	None	None	12
23	V			I			I			None	None	18
24	V		V	I		I	I		I	Hypoesthesia	None	32
25	V			II			II			Paresthesia	None	21
26	V	I		I	CR		I	CR		Paresthesia	None	24
27	V			I			I			Paresthesia	Paresthesia	6
28	V			I			II			None	None	50
29	III			II			II			Paresthesia, Hearing impairment	Contralateral TN (6 months)	12
30		IV			PR			PR		Vertigo, Tinnitus, Persistence of HFS	Persistence of HFS	28
31	V	II		I	PR		I	PR		Paresthesia, Persistence of HFS	Persistence of HFS	47
32	IV		IV	III		I	IV		I	Persistence of TN	Persistence of TN	36
33		IV			CR			CR		Grade I FP	Hearing impairment	12
34	V			I			I			Paresthesia	None	25
35	V			I			I			Hearing impairment	None	48
36		II			PR			NR		Grade III FP	Grade IV FP, Recurrence (2 months)	24
37	IV			I			I			Paresthesia	None	19
38			V			I			I	Hearing impairment	Hearing impairment	48
39	III			I			I			Paresthesia	None	24
40	IV			I			II			None	None	46
41	IV		V	I		I	I		I	None	None	41
42		IV			CR			CR		Grade I FP, Hearing impairment, Tinnitus	Hearing impairment	40
43	V			I			I			None	None	35
44	V			I			I			Grade II FP	Grade II FP	18
45	V			I			I			Grade I FP, Hearing impairment, Diplopia	Hearing impairment	9
46	IV			I			I			CSF fistula, Hypoesthesia	None	22
47	V			I			IV			Diplopia	Recurrence (23 months)	36
48		II			CR			CR		Grade II FP, Tinnitus	Hearing impairment	27
49	V			I			I			None	None	18
50	IV			I			II			Hypoesthesia	None	15
51	V			I			I			None	None	26
52		II			CR			CR		Grade II FP, Conjunctival injection, Epiphora, Hearing impairment	Grade II FP, Unilateral anacusis	12
53	IV			I			I			Hypoesthesia, Hearing impairment	None	9
54	IV			I			I			Hypoesthesia, Tinnitus	None	24
55		III			PR			CR		Grade I FP, Hearing impairment	None	21
56	IV			I			I			Grade II FP, Hypoesthesia, Hearing impairment	Grade II FP	24
57	V			I			I			Paresthesia	None	18
58	IV		IV	I		I	I		I	Hypoesthesia	None	18
59	V			I			I			Grade I FP, Hypoesthesia	None	15
60	IV		V	I		I	I		I	Dysgeusia	None	12
61	V			I			I			Grade I FP, Dysgeusia	Grade I FP	12
62	V			I			I			Hypoesthesia, Hearing impairment	None	12
63	V			I			I			Hypoesthesia	None	12
64	IV			I			I			Hypoesthesia, Tinnitus	None	9
65	V			I			I			Paresthesia, Ear fullness	None	9
66	V			I			I			None	None	6
67	V			I			I			Hypoesthesia	Hearing impairment	6
68	V			I			I			Hypoesthesia	None	6

BNI, Barrow Neurological Institute pain intensity scale; SMC, Samsung Medical Center hemifacial spasm grading system; CR, complete relief; PR, partial relief; NR, no relief; FP, facial paralysis (House-Brackmann grade).

**Figure 7:**
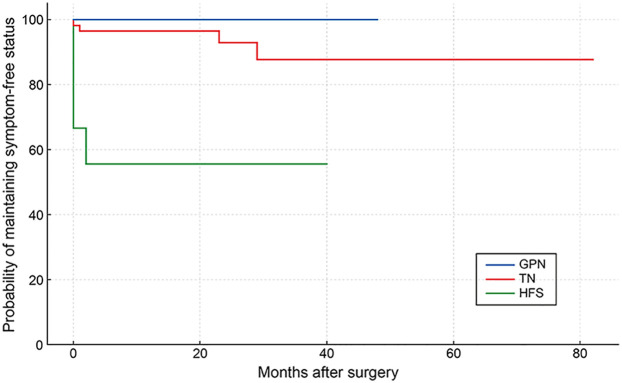
Symptom-free survival in VBD-related NVCS. Kaplan–Meier curves comparing symptom control of TN (red line), HFS (green line) and GPN (blue line) after MVD for VBD. Events were defined as symptom recurrence or persistence (lack of initial complete relief counted at time zero). Patients without events were censored at the last follow-up.

To evaluate the durability of symptom relief after surgery, Kaplan–Meier curves were estimated for TN, HFS, and GPN, and groups were compared with pairwise log-rank tests. The HFS group displayed a clear early step-down in the curve, consistent with persistence of symptoms and additional events thereafter, resulting in significantly poorer survival compared with TN (*p* < 0.001) and GPN (*p* = 0.020). The TN curve showed high survival with only modest late drops, whereas the GPN curve remained stable throughout follow-up; no significant difference was observed between TN and GPN (*p* = 0.318). The median event-free survival could not be estimated for TN or GPN, while in HFS, early failures were evident followed by a subsequent plateau (Figure 7).

### 3.5 Transient and persistent postoperative complications

Immediate postoperative transient complications were observed in 46 patients (67.6%). The most frequent were hearing impairment (37%), facial paralysis (30.4%), hypoesthesia (28.3%), paresthesia (23.9%), ocular impairment (10.9%), and other complications (13%). All transient complications resolved within six months with appropriate treatment. In contrast, persistent deficits were reported in 25 patients (36.8%), including hearing impairment (40%), facial paralysis (24%), paresthesia (4%), dysphonia (4%), and xerophthalmia (4%) (Table 3).

### 3.6 Comparative analysis between VBD-related and classical cases

The VBD group (*n* = 68) was compared with a cohort of 1,066 patients with the classical form of the same NVCS (TN, HFS, GPN) treated in the corresponding period. The mean age at surgery was significantly higher in the VBD patients (60.5 ± 12.1 years) compared to the classical group (55.2 ± 13.6 years; *p* < 0.001). No significant difference was found in symptom duration between the two groups (8.0 ± 6.1 vs. 7.9 ± 6.8 years; *p* = 0.619). The female-to-male ratio differed notably: males predominated in the VBD group, whereas females were more common in the classical group (*p* < 0.001). Likewise, right-to-left side involvement showed a significant difference, left-sided symptoms were significantly more prevalent in the VBD group, whereas right-sided predominance was observed in classical cases (*p* < 0.001). Although the incidence of bilateral cases was less frequent in the VBD group (1.5%) than in the classical group (7.6%), this difference was not statistically significant (*p* = 0.054). No significant differences were found in the incidence of combined neuropathies (11.8% vs. 6.8%; *p* = 0.137) or familial cases (1.5% vs. 2.3%; *p* = 1.000).

Mean surgery duration was significantly longer in the VBD patients (162.4 ± 47.5 min) compared to the classical group (132.9 ± 42.0 min; *p* < 0.001). Regarding comorbidities, hypertension was markedly more prevalent in the VBD cases (58.8% vs. 32.4%; *p* < 0.001), as was diabetes mellitus (30.9% vs. 10.3%; *p* = 0.014). No significant difference was observed in dyslipidemia rates (20.6% vs. 24.0%; *p* =  0.659) (Table 4).

**Table 4:** Comparative analysis between NVCS associated with VBD and classical cases.

Cases	NVCS secondary to VBA	Classical NVCS	*p-*value
	68	1,066	
Age at surgery (yrs)	60.5 ± 12.1	55.2 ± 13.6	**<0.001**
Symptom duration (yrs)	8.0 ± 6.1	7.9 ± 6.8	0.619
M:F ratio	43:25 (1.7:1)	334:732 (0.45:1)	**<0.001**
R:L ratio	28:39 (1:1.4)	606:379 (1:0.6)	**<0.001**
Bilateral HDS	1 (1.5%)	81 (7.6%)	0.054
Combined HDS	8 (11.8%)	72 (6.8%)	0.137
Familiar HDS	1 (1.5%)	24 (2.3%)	1
Surgery time (min)	162.4 ± 47.5	132.9 ± 42.0	**<0.001**
Comorbidities			
Hypertension	40 (58.8%)	345 (32.4%)	**<0.001**
Diabetes mellitus	21 (30.9%)	110 (10.3%)	**0.014**
Dyslipidemia	14 (20.6%)	256 (24.0%)	0.659

NVCS, neurovascular compression syndromes; VBD, vertebrobasilar dolichoectasia; MVD, microvascular decompression. Data are presented as mean ± standard deviation for continuous variables and as number (%) for categorical variables. Student's *t*-test or Fisher's exact test was used as appropriate. Bold values indicate statistical significance.

## 4 Discussion

### 4.1 Demographics and clinical characteristics

The clinical manifestations of VBD include compression of brainstem and cranial nerves, ischemic stroke, cerebral hemorrhage, and hydrocephalus (13). In this retrospective study we reviewed the clinical characteristics, surgical findings, and outcomes of patients with NVCS caused by VBD. The incidence of VBD-associated NVCS in our series was 6%, which falls within the range reported in previous studies (2%–7.7%) (1, 5, 6, 13, 14). Interestingly, a study conducted in a Japanese population reported an incidence of asymptomatic VBD in 1.3% of individuals undergoing routine MRI and MRA (15). Furthermore, autopsy findings suggest that the overall incidence of VBD in the general population is less than 0.05% (13).

The pathogenesis of VBD remains unclear. Its etiology is thought to involve congenital predisposition, infections, immune-mediated mechanisms, and degenerative processes (16). Notably, two of our patients with TN secondary to VBD were siblings who also reported an older sister affected by TN, lending support to a congenital origin. Although combined and bilateral cases were more frequent in the classical group, the differences were not statistically significant. Previous studies have reported that patients with ipsilateral coexistence of HFS and TN often present with a narrow posterior fossa and a large, looped VBA as the offending vessel (17). We observed two cases of combined TN-HFS and six with the coexistence of TN-GPN, whose etiology may be attributed to similar anatomical factors. Moreover, patients with VBD were significantly older at the time of surgery, which may reflect a progressive nature of dolichoectatic changes. Despite comparable symptom durations between groups, the older age of VBD patients may contribute to the higher prevalence of comorbidities such as hypertension and diabetes.

A notable male predominance was observed in the VBD group, in contrast to the female predominance seen in classical cases. Additionally, left-sided symptoms were more frequent among VBD patients, whereas right-sided involvement predominated in the classical group. These findings are consistent with previous reports of male and left-sided predominance in VBD-induced neurovascular compression and may reflect underlying anatomical or hemodynamic variations (6, 8, 18, 19). The left-sided predominance may be attributed to anatomical asymmetry of the vertebral arteries and hemodynamic factors. The left vertebral artery, which arises directly from the aortic arch, typically carries greater blood flow and experiences higher shear stress compared to the right, which originates from the brachiocephalic trunk. This asymmetry results in uneven blood flow to the basilar artery, contributing to elongation and curvature of the VBA complex toward the weaker vertebral artery (6, 7). Additionally, in most of our patients with TN, pain was distributed along the V2 and V3 dermatomes, which aligns with the somatotopic organization of sensory fibers in the trigeminal REZ (5).

Hypertension emerged as a significant comorbidity in our cases and previous reports, supporting the association between elevated blood pressure and tortuosity of the vertebrobasilar system (7, 18). However, the physiological basis of this relationship is not yet fully understood. It has been proposed that the hemodynamic effects of elevated blood flow through an atherosclerotic vessel may lead to tortuosity. Conversely, it has also been suggested that compression of the ventrolateral medulla by tortuous vessels may contribute to the development of hypertension and potentially alter cerebral blood flow (20). Regardless of the underlying cause of hypertension, perioperative blood pressure control is essential to minimize stroke risk (13).

Diabetes mellitus was another comorbidity with a higher incidence among VBD cases. It has been identified as a risk factor for the development of classical TN, possibly due to hyperglycemia-induced nerve injury, which may represent the underlying pathophysiological link between these two conditions (21). Moreover, morbid obesity combined with diabetes has been associated with an increased risk of MVD reoperation (22); however, in our series, reoperated patients presented only with overweight or grade I obesity. Finally, dyslipidemia has also been reported as a potential risk factor for both TN and HFS (23).

Preoperative high-resolution MRI is essential not only for ruling out alternative etiologies, such as malformations, neoplasms, or cerebrovascular diseases, but also for surgical planning (5, 24). However, when MRIs were interpreted by external radiologists, VBA involvement was recognized in only 63.2% of cases, despite the prominent caliber of the artery. The lower sensitivity likely reflects that general neuroradiologists are usually less experienced in identifying neurovascular conflicts. In contrast, neuroradiologists at our institution, who are accustomed to evaluating vascular-related compression, achieved a 100% detection rate, indicating that systematic training and focused assessment improve diagnostic accuracy. Therefore, it is recommended to complement MRI with angiographic studies to allow precise assessment of vessel elongation, tortuosity, and enlargement.

### 4.2 Surgical findings

The intraoperative findings underscored the complexity of VBD-induced neurovascular conflicts. In 50% of our cases, the VBA was the sole offending vessel; in the remaining cases, additional arterial or mixed compressions were present. The most commonly associated arteries were the SCA and the AICA, consistent with previous studies (7, 19, 24).

According to Szapiro's classification, most patients exhibited grade II or grade III compression by the VBA, this significant proportion of complex cases highlights the technical challenges associated with this patient cohort (9). On the other hand, arachnoid adhesions were found in the majority of patients, further complicating surgical manipulations. Although the origin of these arachnoid alterations remains uncertain, chronic inflammation has been suggested as a potential contributing factor (25). Additionally, the incidental discovery of atherosclerotic plaques represents a significant surgical concern, as these lesions may rupture during mobilization, potentially leading to embolic events such as ischemic stroke or intracerebral hemorrhage (5). To minimize these risks, mobilization and transposition should be performed on a non-atherosclerotic segment of the VBA whenever possible. Another incidental finding was the presence of prominent suprameatal tubercles, which required careful drilling and prolonged surgical manipulation. Even excluding all incidental findings, decompression of a large and tortuous vessel was associated with significantly longer operative times compared to MVD performed for classical cases. These observations underscore the critical importance of meticulous preoperative planning to anticipate intraoperative difficulties that may influence both the surgical strategy and patient outcomes.

### 4.3 Treatment results

Postoperative outcomes were favorable, with a high proportion of patients achieving complete pain relief, consistent with other reports on the effectiveness of MVD in VBD-associated cases (6, 8). Although recurrence rates were low, they did occur within a range of months to several years postoperatively, highlighting the importance of long-term follow-up (26). In the patient who experienced recurrence and underwent reoperation, granuloma formation was identified (Figure 4E). Since granulomas are a known cause of symptom recurrence, their surgical removal and re-exploration remain the recommended treatment approach (5).

The median follow-up period of 27.3 months revealed sustained symptom-free status in most cases. These outcomes are comparable to those reported in the literature, supporting the long-term efficacy of MVD for VBD-related NVCS (7, 8). Notably, the patient who experienced recurrence presented with evident VBA atherosclerosis during both surgical procedures, suggesting a potential association between this vascular pathology and pain recurrence.

In the Kaplan–Meier analysis, the HFS curve shows an early step-down, indicating that early failure, rather than recurrence, drives its inferior outcomes after VBD. By contrast, TN maintained high event-free survival with only modest late declines, and GPN remained nearly flat, consistent with durable symptom control once operated. Clinically, these findings support preoperative counseling that highlights a higher risk of early failure in HFS, while reinforcing that TN and GPN generally achieve durable relief when decompression is adequate. Limitations of this analysis include the small size of the HFS and GPN subgroups and the low number of events, while differences in follow-up, baseline anatomy, or technique selection may also have influenced the results.

Alternative treatment options for NVCS may be appropriate in selected patients and include percutaneous neuroablative procedures (radiofrequency thermocoagulation, glycerol rhizotomy, and balloon compression) as well as radiosurgery. However, these modalities do not address the underlying mechanism of VBD-related compression and may be associated with sensory deficits or suboptimal therapeutic responses (27–30). In our cohort (characterized by older age, comorbidities, and frequent mixed conflicts), MVD provided immediate etiologic relief with an acceptable safety profile, in line with contemporary series (6, 24, 26, 31).

### 4.4 Postoperative complications

Most postoperative deficits were successfully managed with conservative treatment and resolved within approximately six months. The most frequent persistent complications, such as hearing impairment and facial paralysis, reflect the inherent risks of manipulating a rigid and tortuous VBA (18, 19). Despite the thin and fragile nature of the VBA wall, no deaths have been reported due to intraoperative rupture of the vessel (32).

Although several studies have compared the safety, efficacy, and complication rates of interposition and transposition techniques for MVD in VBD-related NVCS (6, 31, 33–37), we primarily opted for the interposition method. Some authors report that both techniques offer similar safety and efficacy profiles (6, 31, 36, 37), while others suggest that interposition is associated with a lower rate of long-term complications (6), and may achieve earlier resolution of spasms (35). Conversely, transposition has been proposed to provide superior long-term pain relief (33), a lower incidence cranial nerve dysfunction, reduced risk of implant displacement, and avoidance of granuloma formation (6, 14, 34). However, transposition requires a wider surgical corridor, longer operative time, and involves increased manipulation, which may lead to VBA occlusion, injury, or vasospasm of small arteries. Additionally, the presence of atherosclerotic plaques further increases the risk, as their rupture during mobilization may result in embolic events leading to ischemic stroke or intracranial hemorrhage (5). Ultimately, the selection of the appropriate technique should consider patient-specific factors and the surgeon's experience.

### 4.5 Limitations of the study

Our study has several limitations. The sample size was relatively small for HFS, GPN, and combined neuropathies, which restricted the Kaplan–Meier analysis and further limits the generalizability of the findings. The retrospective, single-center design may have introduced selection and observational biases. Additionally, the relatively short postoperative follow-up in some patients may have hindered the full characterization of long-term outcomes. Despite these limitations, the study provides valuable insight into the surgical management of NVCS caused by VBD and may contribute to the optimization of surgical outcomes in this complex population. To strengthen the evidence base, randomized prospective studies and multicenter collaborations are warranted to overcome sample size limitations and to determine the optimal surgical strategy for VBD-related NVCS.

## 5 Conclusion

This study highlights the challenges and successes in managing NVCS caused by VBD through MVD. The findings underscore the critical importance of surgical experience, meticulous preoperative planning, and long-term follow-up in achieving favorable outcomes. Although complications remain a concern, the overall safety and efficacy of MVD in this patient subset reaffirm its role as the gold standard for treating refractory NVCS. Future research should aim to refine surgical techniques and optimize perioperative management to further enhance outcomes in this challenging patient population.

## Data Availability

The raw data supporting the conclusions of this article will be made available by the authors, without undue reservation.
